# Supplementary material for: Superdiffusion dominates intracellular particle motion in the supercrowded cytoplasm of pathogenic Acanthamoeba castellanii
Source: Sci Rep. 2015 Jun 30;5:11690. doi: 10.1038/srep11690 (PMC5155589; doi:10.1038/srep11690)
Supplement: Supplementary Information [file srep11690-s1.pdf]

Supplementary information for:

Superdiffusion dominates intracellular particle motion in the supercrowded cytoplasm of pathogenic *Acanthamoeba castellanii*

Julia F. Reverey, Jae-Hyung Jeon, Han Bao, Matthias Leippe, Ralf Metzler, Christine Selhuber-Unkel

Displacement distribution for the intracellular motion

Fig. S1 presents the displacement distributions, $P(x,t)$ and $P(y,t)$, for the intracellular motion of normal acanthamoebae. Here, the intracellular motion $\vec{r}_I(t)$ is a drift-free part in the tracked trajectory $\vec{r}(t)$, obtained from the $\vec{r}(t)$ by subtracting the centroid contribution from it. In the figure the displacement distributions at several times are plotted together with the Gaussian fit to the data. The results show that the intracellular motion reasonably follows Gaussian statistics, albeit the distributions are noisy at larger t. Given the fact that the intracellular motion $\vec{r}_I(t)$ is superdiffusive (or subdiffusive) in the experiments, this supports the validity of our simple model describing the intracellular motion in the frame of fractional Brownian motion (i.e., the Gaussian anomalous diffusion process).[1]

Velocity autocorrelation function (VACF)

In this section we explain in detail the velocity autocorrelation function considered in our study with additional supplementary plots. Assume that the particle motion $\vec{r}(t)$ tracked by video tracking is the superposition of the drift of acanthamoeba during crawling $\vec{V}_d$ and the intrinsic intracellular motion $\vec{r}_I$ such that

$$\vec{r}(t) = \vec{V}_d t + \vec{r}_I(t). \tag{S1}$$

The internal motion $\vec{r}_I(t)$ explains the net motion of a particle inside the acanthamoeba that arises potentially from its passive diffusion and/or the active transport by motor proteins. Given an arbitrary time interval δt we define an average velocity for the tracked trajectory $\vec{r}(t)$

$$\vec{V}_{\delta t}(t) = \frac{\vec{r}(t+\delta t) - \vec{r}(t)}{\delta t}. \tag{S2}$$

Since the shortest time interval in the experiment is the unit video frame interval $t_0 \approx 0.01$ s and the particle motion occurs in the overdamped limit at this time scale, one should keep in mind that the above velocity defined from the overdamped motion is not the instantaneous velocity $\vec{V}(t)$ defined in the underdamped limit. As seen below in Supplementary Equation (S3), the square of the velocity in the overdamped limit $\langle \vec{V}_{\delta t}^2(t)\rangle = \langle \vec{r}^2(\delta t)\rangle/\delta t^2$ is a quantity related to the mean square displacement while the instantaneous velocity in the underdamped limit gives the relation $\vec{V}^2(t) \sim k_B\mathscr{T}$. In the former case the effect of temperature exists in the parameters such as diffusion constant and the diffusion exponent. The velocity autocorrelation function (VACF) is conventionally obtained from the ensemble-average of many trajectories. Using the velocity defined in Supplementary Equation (S2) the VACF $C(\Delta) = \langle \vec{V}_{\delta t}(\Delta+t)\cdot\vec{V}_{\delta t}(t)\rangle$ is given by

$$\begin{aligned} C(\Delta) &= \{C_{rr}(\Delta+t+\delta t, t+\delta t) + C_{rr}(\Delta+t, t) - C_{rr}(\Delta+t+\delta t, t) \\ &- C_{rr}(\Delta+t, t+\delta t)\}/\delta t^2, \end{aligned} \tag{S3}$$

where $C_{rr}(t_1,t_2) = \langle \vec{r}(t_1)\cdot\vec{r}(t_2)\rangle$ is the covariance of the position vector. Instead of this, alternatively, we obtain an individual VACF curve from a single trajectory by the definition

$$C(\Delta) = \overline{\vec{V}_{\delta t}(t+\Delta)\cdot\vec{V}_{\delta t}(t)} = \frac{1}{T-\Delta-\delta t}\int_0^{T-\Delta-\delta t} dt \vec{V}_{\delta t}(t+\Delta)\cdot\vec{V}_{\delta t}(t). \tag{S4}$$

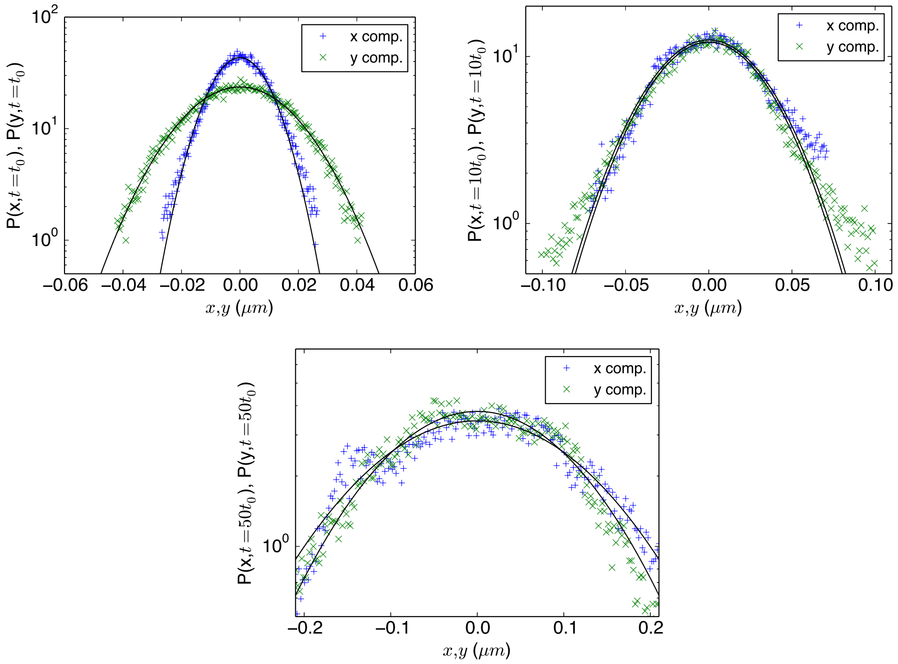

Supplementary Figure S1. Displacement distributions of the intracellular motion $\vec{r}_I(t)$ for normal acanthamoebae. Distributions $P(x,t),\ P(y,t)$ for x- and y-component at $t=t_0$ (upper left), $10t_0$(upper right), and $50t_0$ (bottom), where $t_0 \approx 0.01$ sec is the time interval between video frames. Solid lines represent the Gaussian fit to the result.

For the ergodic diffusive processes considered in our study, we expect

$$\overline{\vec{V}_{\delta t}(t+\Delta)\cdot\vec{V}_{\delta t}(t)} = \langle \vec{V}_{\delta t}(t+\Delta)\cdot\vec{V}_{\delta t}(t)\rangle.$$

Thus, the time-averaged VACF obtained from Supplementary Equation (S4) should be the same as the conventional VACF, if all the tracked particles have the identical diffusion behavior. However, intracellular diffusion is typically heterogeneous and thus the time-averaged VACFs can have trajectory-to-trajectory variations.

Model

Now we model the intracellular motion $\vec{r}_I(t) = (x_I(t), y_I(t))$ to follow fractional Brownian motion (FBM).[1] That is, each component motion in $\vec{r}_I(t)$ is a correlated Gaussian processes characterized by the mean $\langle x_I(t)\rangle = \langle y_I(t)\rangle = 0$ and the covariance $C_{xx}(t_1,t_2) = C_{yy}(t_1,t_2) = K_\alpha(|t_1|^\alpha + |t_2|^\alpha - |t_1-t_2|^\alpha)$ with the exponent $0<\alpha<2$.[1] From the mean squared displacement $\langle \vec{r}_I^{\,2}(t)\rangle = 4K_\alpha t^\alpha$, FBM is classified to subdiffusion for $0<\alpha<1$, diffusive for $\alpha=1$, and superdiffusive for $1<\alpha<2$. Using this statistical property we obtain the normalized velocity autocorrelation function

$$\frac{C(\Delta)}{C(0)} = \frac{V_d^2 + \langle V_{\delta t}^I(t) V_{\delta t}^I(0)\rangle}{V_d^2 + \langle [V_{\delta t}^I(0)]^2\rangle}, \tag{S5}$$

$$= \frac{V_d^2/4K_\alpha(\delta t)^{\alpha-2} + [(t/\delta t+1)^\alpha + |t/\delta t - 1|^\alpha - 2(t/\delta t)^\alpha]/2}{V_d^2/4K_\alpha(\delta t)^{\alpha-2}+1}. \tag{S6}$$

In this expression $V_d = |\vec{V}_d|$ and $\vec{V}_{\delta t}^I(t) = [\vec{r}_I(t+\delta t) - \vec{r}_I(t)]/\delta t$ denotes the average velocity for the intracellular motion $\vec{r}_I(t)$. We note that the relaxation profile of $C(\Delta)$ is fully governed by the VACF of the intracellular part $C^I(\Delta) = \langle \vec{V}_{\delta t}^I(\Delta+t)\cdot\vec{V}_{\delta t}^I(t)\rangle$.
Effect of α: The main advantage of studying $C(\Delta)$ is that one obtains a basic information about the stochastic identity of the intracellular motion $\vec{r}_I(t)$ from the tracked position $\vec{r}(t)$ without extracting $\vec{r}_I$ from it. As we plot in Fig. 4 in the main text, $C(\Delta)$ has three distinguished profiles depending on the range of α: (1) For subdiffusive intracellular motion with $0<\alpha<1$ the VACF has a dip at $t=\delta t$, owing to the anti-persistent intracellular motion, and then relaxes towards the positive saturation value. (2) If the intracellular motion is a normal random walk with $\alpha=1$ the VACF has no relaxation for $t\geq\delta t$. (3) If the intracellular motion is superdiffusive with $1<\alpha<2$ the VACF is always positive at all times and monotonically decreases from unity to the saturation value.

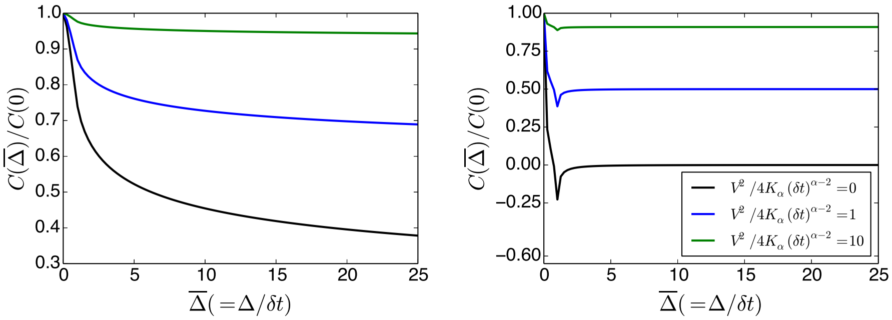

Supplementary Figure S2. Profiles of the theoretical VACF curves (S6) at varying the ratio $V_d^2/4K_\alpha(\delta t)^{\alpha-2}$. The VACFs are plotted against the rescaled lag time $\Delta/\delta t$. (Left) Superdiffusive intracellular motion $\vec{r}_I(t)$ with $\alpha = 1.8$. (Right) Subdiffusive intracellular motion $\vec{r}_I(t)$ with $\alpha = 0.2$.

Effect of the amoeba drift and time interval (V_d, δt, and K_α): As the above VACF (S6) shows, the profile and the saturation value ($V_d^2/[V_d^2 + 2K_\alpha(\delta t)^{\alpha-2}]$) of the VACF depend on the time interval δt and the exponent α as well as V_d. Physically, such dependences are plausible; In Supplementary Equation (S6) the term $V_d^2/4K_\alpha(\delta t)^{\alpha-2}$ is the ratio between the two square displacements accomplished by the acanthamoeba's drift $(V_d\delta t)^2$ and by the intracellular transport $4K_\alpha(\delta t)^\alpha$ over the time interval δt. In case this term is large, the effect of the acanthamoeba's drift dominates over the intracellular contribution. In the opposite situation where the acanthamoeba's drift is negligible (as we observed in the blebbistatin-treated amoeba), the VACF $C(\Delta)$ is dominated by the VACF of the intracellular motion. In Supplementary Fig. S2 we plot the variation of VACF profiles against the change of these parameters for both superdiffusive and subdiffusive intracellular motions with the exponent α found in our experiment. Note that each curve converges to its own saturation value $V_d^2/[V_d^2 + 2K_\alpha(\delta t)^{\alpha-2}]$.

Experiment

In the main text we present VACF curves for $x(t)$ for the normal and drug-treated acanthamoeba using equation (S4) with $\delta t = 10t_0$. Here we provide additional VACF curves.

Time intervals: In Supplementary Fig. S3 we plot the same VACF curves (shown in the main text) for the normal, blebbistatin-, latrunculin A-, and nocodazole-treated acanthamoeba at various time intervals $\delta t = t_0$, $5t_0$, $10t_0$, and $20t_0$. For comparison, the VACFs are plotted against the rescaled lag time $\overline{\Delta}(= \Delta/\delta t)$ because the $C(\Delta)$ is always self-correlated up to $\Delta = \delta t$ and then the regime for $\Delta > \delta t$ tells about the correlation of a velocity between two time points. Note that at a given $\overline{\Delta}$ the number of data points plotted in the VACF of $\delta t = nt_0$ is n times that of the VACF of $\delta t = t_0$. Because of this, for all the plotted VACFs always the one with $\delta t = t_0$ has the largest fluctuation and varies in a discontinuous manner. The results show that except for the case of $\delta t = t_0$ the rest VACFs exhibit consistent patterns. In fact in our study the VACF with $\delta t = t_0$ is not a proper choice in studying superdiffusive intracellular motion observed in the experiment. Different from the above theoretical model based on fractional Brownian motion, the experimentally tracked particle motion has a time-dependent diffusion exponent $\alpha = \alpha(\Delta)$. As TA MSD curves (Figs. 3, 5, 7 and 8 in the main text) for the tracked particles show, the superdiffusive motion is observed only for $\Delta \geq O(10t_0) \approx 0.1$ (sec). At $\Delta = t_0$ the TA MSD curves exhibit a pattern of strong subdiffusive motion. Therefore at this shortest time interval (where the effect of the acanthamoeba drift motion is minimal) the VACFs have patterns clearly distinguished from the profile for superdiffusive motion. For our study the time interval of $\delta t = 10t_0$ seems to be a good choice for analyzing the superdiffusive intracellular motion while reducing the effect of the acanthamoeba drift in the VACF curve. Note that with our choice of $\delta t = 10t_0$ the ratio $V_d^2/4K_\alpha(\delta t)^{\alpha-2}$ safely lies in the range [0, 1] for all the experiments presented in this study. **VACF curves for the intracellular motion $\vec{r}_I(t)$:** As supplement figures we plot in Supplementary Fig. S4 the VACF curves $C^I(\Delta)/C^I(0)$ for the intracellular motion $\vec{r}_I(t)$, which is the trajectory of particles in the frame of the centriod of an acanthamoeba. Because the acanthamoeba is not a rigid body, all the relative trajectories from the centroid may not correspond to the true intracellular motion. Nevertheless our approach seems to be fairly reasonable, as seen in this plot; the obtained intracellular part $\vec{r}_I(t)$ has a positive correlation in its VACF and its decay converges to zero.

Time-averaged displacement (drift)

To see the trajectory-to-trajectory variation of the particle drift due to the locomotion of the acanthamoeba we calculate the time-averaged displacement

$$\overline{\delta(\Delta)} = \frac{1}{T-\Delta}\int_0^{T-\Delta} dt[x(t+\Delta) - x(t)] \quad \text{(S7)}$$

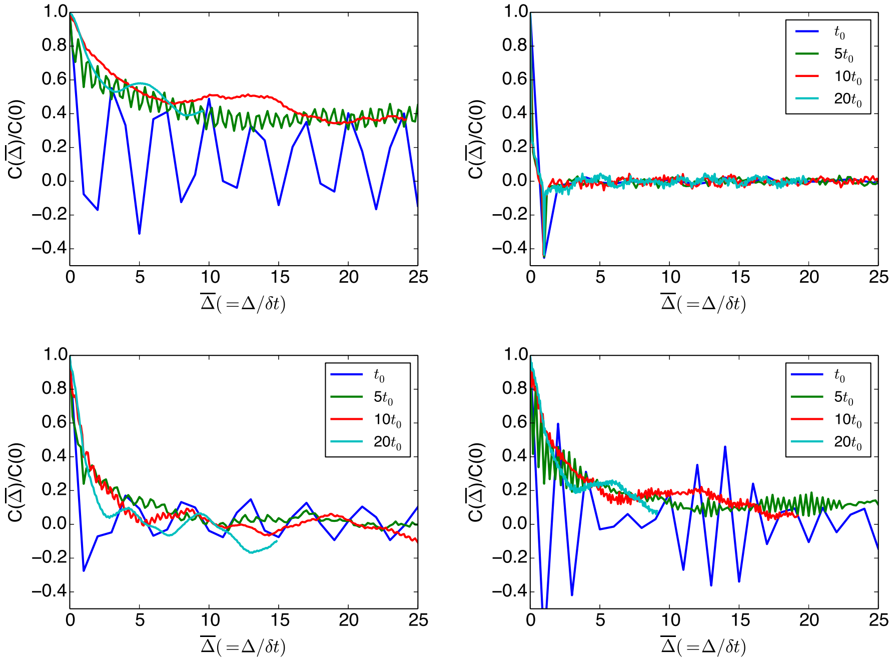

Supplementary Figure S3. Profiles of the experimental VACF curves at $\delta t = t_0,\ 5t_0,\ 10t_0$, and $20t_0$: (Upper left) Untreated acanthamoeba. (Upper right) Blebbistatin-treated acanthamoeba. (Lower left) Latrunculin A-treated acanthamoeba. (Lower right) Nocodazole-treated amoeba. In each curve, one of the corresponding VACF curves presented in the main text is used.

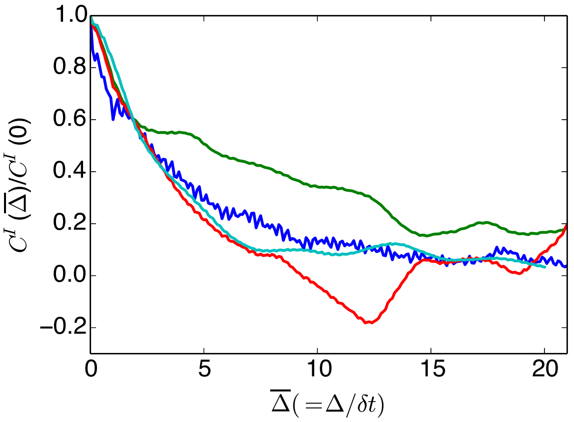

Supplementary Figure S4. Samples of VACF curves $C^I(\overline{\Delta})/C^I(0)$ with $\delta t = 10t_0$ for the intracellular motion. Experimental data are from untreated acanthamoeba.

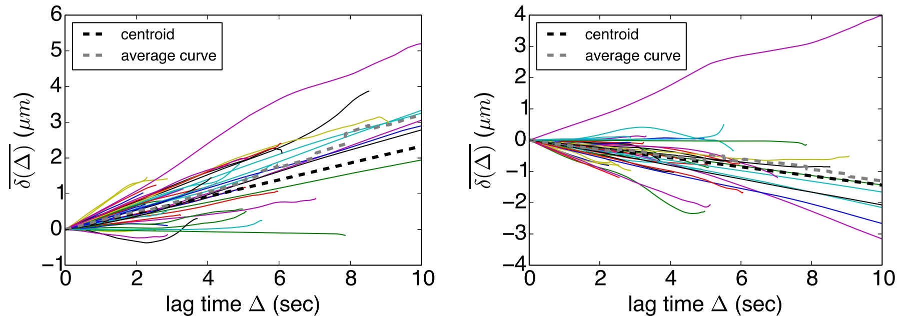

Supplementary Figure S5. Time-averaged displacements $\overline{\delta(\Delta)}$ for about 40 intracellular particles (solid lines) in a moving acanthamoeba. Their average and the centroid motion of the acanthamoeba are also shown. (Left) x-component part. (Right) y-component part.

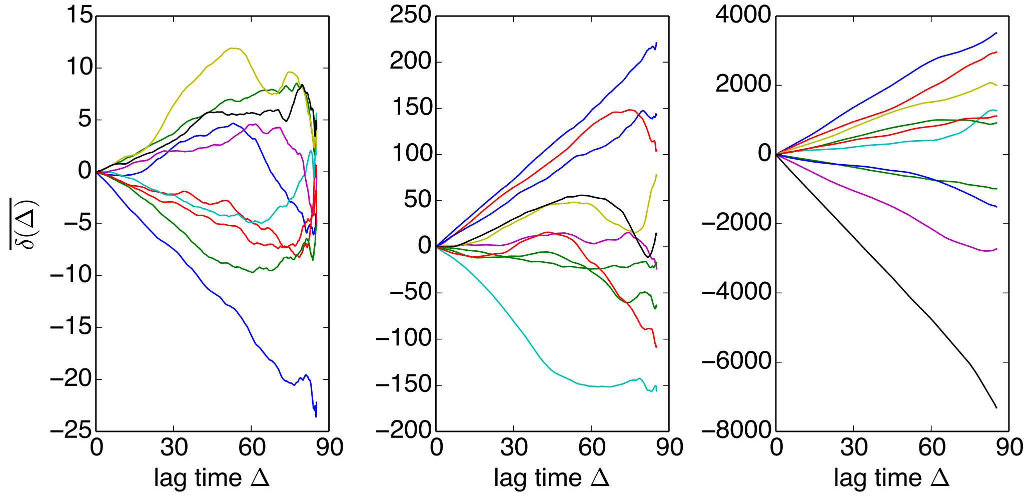

Supplementary Figure S6. Time-averaged displacement $\overline{\delta(\Delta)}$ for 10 simulated FBM trajectories. (Left) $\alpha = 0.5$ (subdiffusion). (Middle) $\alpha = 1.0$ (normal diffusion. (Right) $\alpha = 1.8$ (superdiffusion).

for both x and y coordinate components. Supplementary Figure S5 presents the TA displacement traces for about 40 particles in a moving acanthamoeba. The results are compared with the TA displacement of the acanthamoeba itself via its centroid motion. Note that the averaged trace for the individual particles almost exactly follows the trace for the centroid at lag times $\Delta < 3$ (sec), (above which the disagreement between the two is because only a few trajectories were used for the averaging). This shows that on average the linear increase or decrease of TA displacements with Δ is determined by the locomotion of the acanthamoeba. Then the scatter of individual TA displacements from the average is due to the intracellular particle motion independent from the drift. It is confirmed in Supplementary Fig. S6, in which the TA displacements of FBM with $\alpha = 0.5$ (subdiffusion), 1 (normal), 1.8 (superdiffusion) are depicted. In all cases the TA displacements have symmetric dispersion from the average $\overline{\delta(\Delta)} = 0$.

Further details on the experimental results

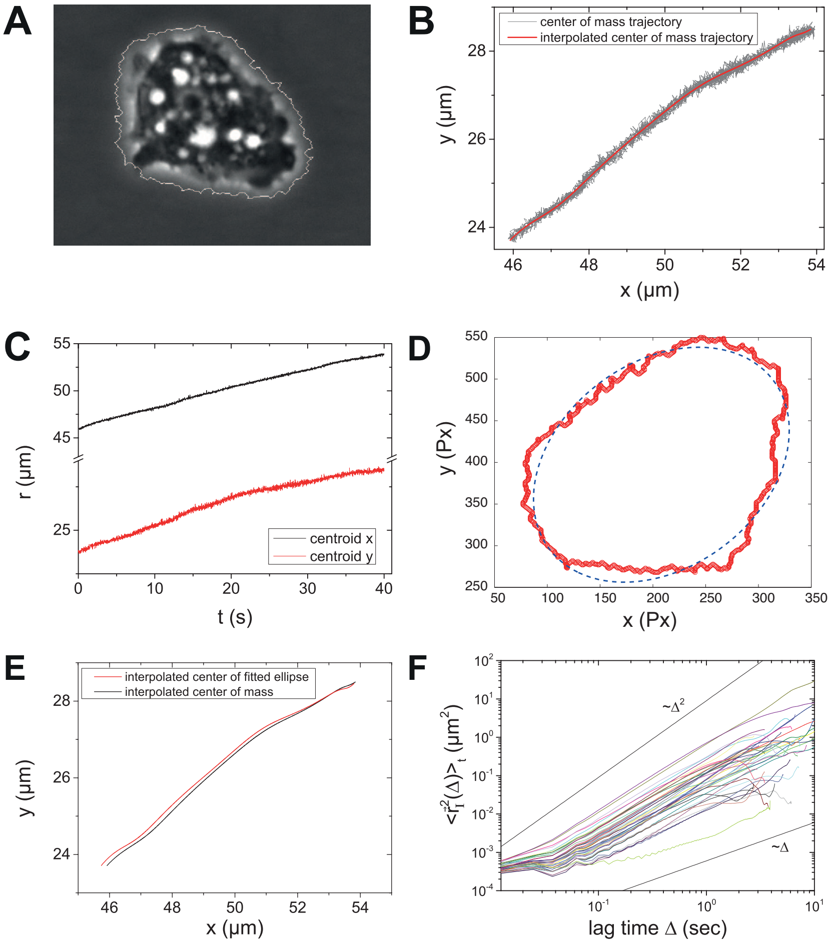

Supplementary Figure S7. Determination of acanthamoeba centroid motion. (A) Representative segmented amoeba. Image segmentation was carried out with a home-written image segmentation algorithm adapted from the example "Detecting a Cell Using Image Segmentation" in the Image Processing Toolbox of MATLAB (The MathWorks, Natick, MA). The centroid was calculated from the segmented cell image assuming a homogeneous mass distribution within the cell boundary. (B) Trajectory (grey) of the centroid of the acanthamoeba (i.e. the center of mass of the segmented acanthamoeba). Due to the phase contrast halo and segmentation artefacts the centroid position got very noisy so that a polynomial fit was used to interpolate the curve (red). This interpolated curve was used for further analyses. (C) x and y position of the centroid (non-interpolated) over time showing the relatively constant speed of acanthamoeba motion. The centroid can in principle be also determined by fitting an ellipse to the amoeba's outline. (D) shows an ellipse fitted using the fitellipse-function MATLAB. It was plotted with the plotellipse-function (fitellipse.m and plotellipse.m: copyright Richard Brown). (E) confirms that there is only a marginal difference in the centroid position determined with the two methods. In (F) we show TA MSDs calculated by correcting trajectories with the centroid determined by fitting the ellipse. There is no significant difference between this MSD plot and the one shown in Fig. 3B.

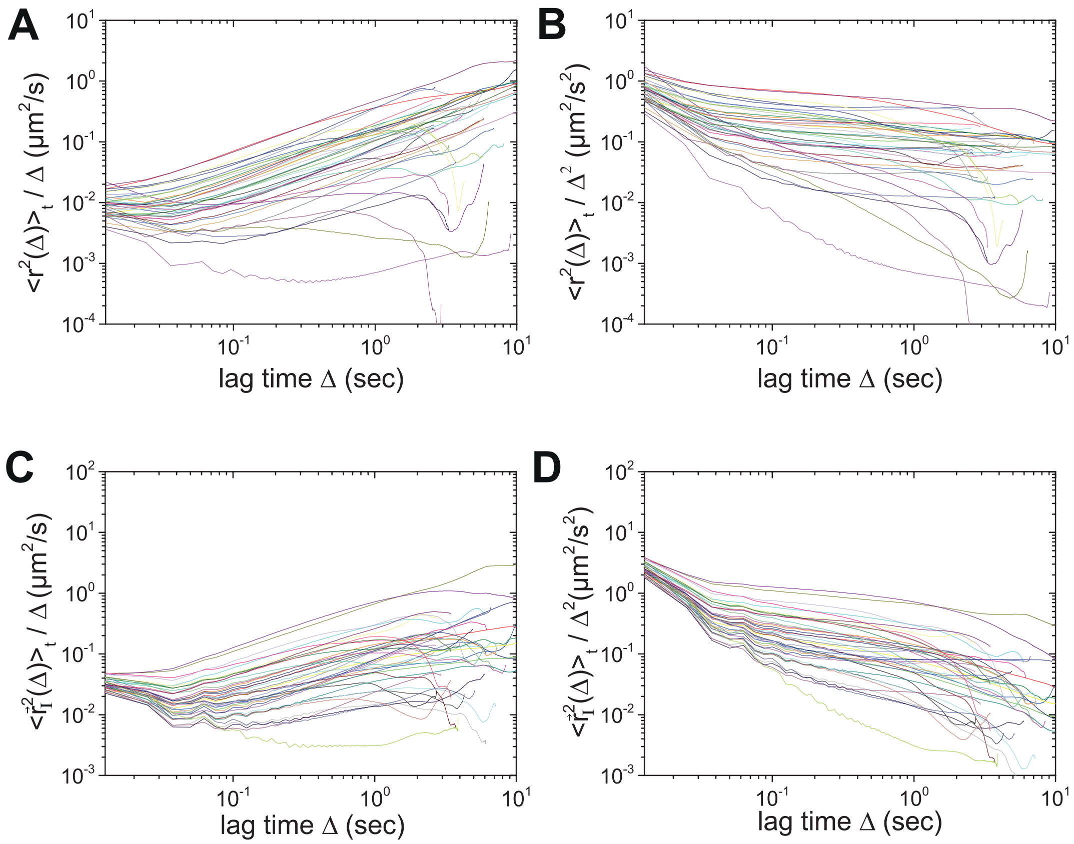

Supplementary Figure S8. TA MSDs divided by lag time Δ(A,C) and Δ^2(B,D). (A) and (B) show TA MSDs calculated from the raw trajectories of untreated acanthamoeba, (C) and (D) were calculated from the same trajectories after centroid correction, i.e. they represent intracellular motion relative to the centroid. Clearly, most of the trajectories represent superdiffusive motion.

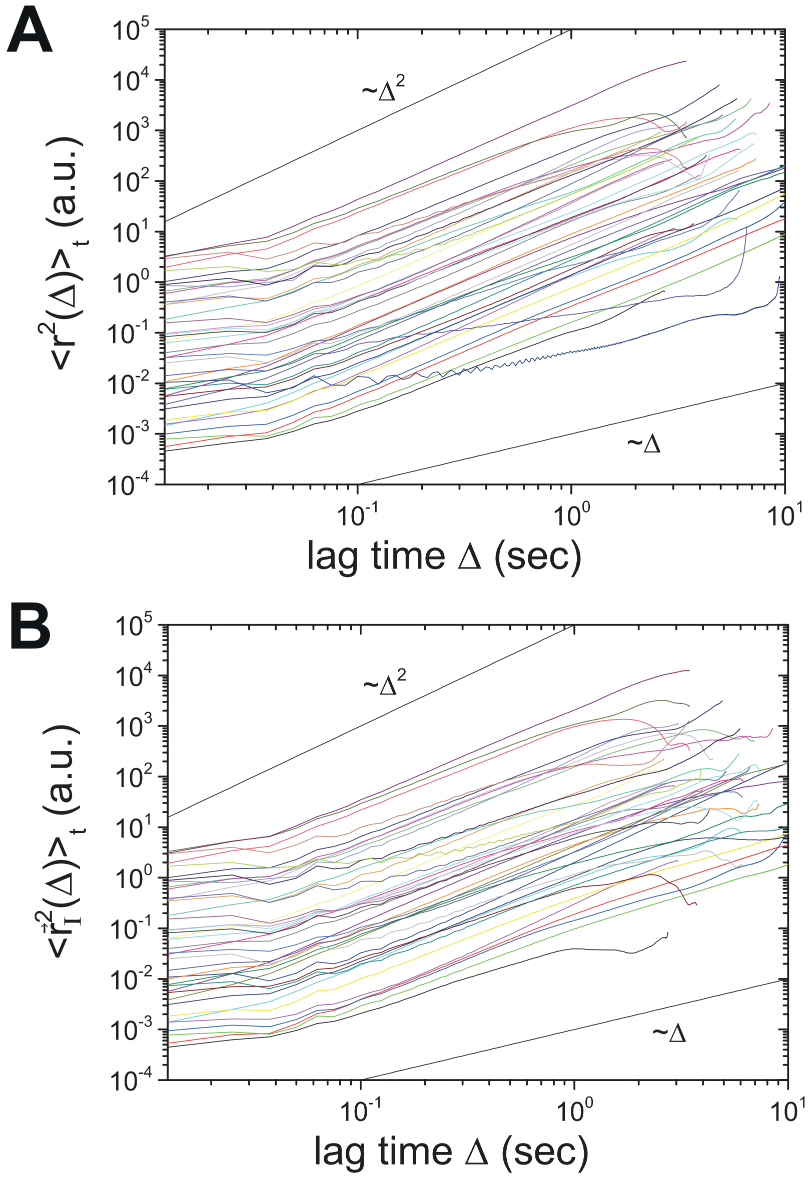

Supplementary Figure S9. Stacked TA MSD plots for better visibility of single trajectories. (A) trajectories of untreated acanthamoebae, (B) trajectories of untreated acanthamoebae relative to the centroid.

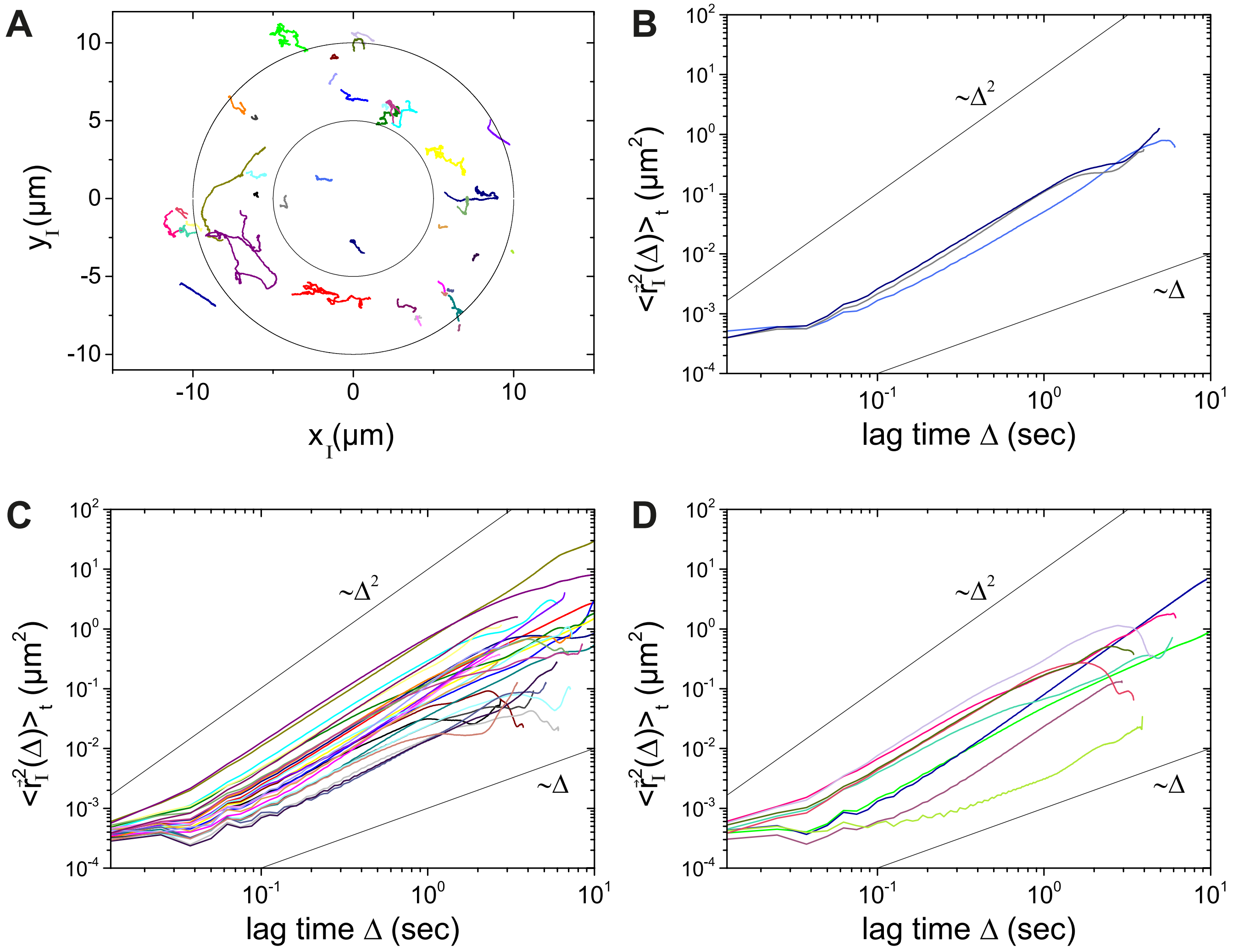

Supplementary Figure S10. TA MSDs as a function of particle position relative to the centroid of the acanthamoeba. (A) Classification of particle position relative to the center of the amoeba. Three zones were generated: a central zone, an intermediate zone, and an outer zone. TA MSDs are shown in (B) for the central zone, in (C) for the intermediate zone, and in (D) for the outer zone.

References

1. Mandelbrot, B. B. & Van Ness, J. W. Fractional Brownian Motions, Fractional Noises and Applications. SIAM Review 10, 422-437, (1968).
